# A recurrence relation for the average singular value of a complex Gaussian random matrix

**DOI:** 10.1007/s11785-026-01988-4

**Published:** 2026-08-07

**Authors:** Luís Daniel Abreu

**Affiliations:** NuHAG, Faculty of Mathematics, University of Vienna, Oskar-Morgenstern-Platz 1, A-1090 Vienna, Austria

**Keywords:** Complex Gaussian matrix, Average singular value

## Abstract

In this brief note, we obtain a recurrence relation for the average singular value α(d) of a complex valued d×d matrix $\frac{1}{\sqrt{d} }X$ with Gaussian entries, of the form:

$$\begin{aligned} \alpha _{ \mathbb {C} }\left( d+1\right) =\alpha _{ \mathbb {C} }\left( d\right) +R(d)\text {,} \end{aligned}$$

where *R*(*d*) can be written as the linear combination of two integrals involving Laguerre polynomials. This reduces to showing that R(d)<0 (a non-trivial analysis problem) a 2016 conjecture of Bandeira, Kennedy and Singer, where it is predicted that α(d) decreases monotonically with *d*, from $\alpha _{ \mathbb {C} }\left( 1\right) =\sqrt{\frac{\pi }{4}}$, to the $d\rightarrow \infty$ limit $\frac{8}{3\pi }$ provided by the Marchenko-Pastur distribution. While this may point to a possible path to solving the conjecture, it is still far from a solution, since the two integrals *R*(*d*) are non-summable hypergeometric functions, and showing that R(d)<0 is a non-trivial analysis problem.

## Introduction

### The average singular value of a complex Gaussian random matrix

Let $$X_{d}$$ be a d×d complex Gaussian random matrix whose random i.i.d. the entries are standard complex Gaussian random variables with density $$\pi ^{-1}e^{-|z|^{2}}$$ on $$\mathbb {C}$$. The matrix $$ W_{d}=d^{-1}X_{d}X_{d}^{*}$$ is usually referred to in the literature as a complex Wishart matrix. If $$x_{1},...x_{d}$$ are the eigenvalues of $$W_{d}$$ , then the singular values of $$d^{-1}X_{d}$$ are

$$\begin{aligned} \sqrt{\frac{x_{1}}{d}},...,\sqrt{\frac{x_{d}}{d}}\text {.} \end{aligned}$$

Let

$$\begin{aligned} s_{k}\left( \frac{1}{\sqrt{d}}X_{d}\right) \end{aligned}$$

denote the *kth* singular value of $\frac{1}{\sqrt{d}}X_{d}$. The corresponding *average singular value* can be written as the expected (bulk, non-ordered) singular value :

$$\begin{aligned} \alpha _{ \mathbb {C} }\left( d\right) =\mathbb {E}\left[ \frac{1}{d}\sum _{k=1}^{d}s_{k}\left( \frac{1}{\sqrt{d}}X_{d}\right) \right] \text {.} \end{aligned}$$

with respect to the empirical singular values measure

$$\begin{aligned} \mu _{\frac{1}{\sqrt{d}}X_{d}}=\frac{1}{d}\sum _{k=1}^{d}\delta _{s_{k}(\frac{ 1}{\sqrt{d}}X_{d})}\text {.} \end{aligned}$$

The asymptotic value of $\alpha _{ \mathbb {C} }\left( d\right)$ when $\alpha \rightarrow \infty$,

1.1 $$\begin{aligned} \lim _{d\rightarrow \infty }\alpha _{ \mathbb {C} }\left( d\right) =\int _{0}^{4}x^{\frac{1}{2}}\frac{1}{2\pi x}\sqrt{x(4-x)}dx= \frac{8}{3\pi }\text {,} \end{aligned}$$

can be obtained from the Marchenko-Pastur [9] quarter-circle distribution,

1.2 $$\begin{aligned} \lim _{d\rightarrow \infty }d\mu _{\frac{1}{\sqrt{d}}X}(x)=\frac{1}{2\pi x} \sqrt{x(4-x)}\textbf{1}_{\left[ 0,4\right] }dx\text {.} \end{aligned}$$

The Marchenko-Pastur law has several applications and triggered an impressive body of research (see, for instance, [16] for applications, and [6] for the asymptotics of extreme values), questions regarding monotonic behavior of the average singular value seem to have remained unexplored until [4, Conjecture 8], where it has been conjectured that $\alpha _{ \mathbb {C} }\left( d\right)$ decreases monotonically when approaching the Marchenko-Pastur limit (1.1). With this conjecture in mind, the author has established the following recurrence relation

1.3 $$\begin{aligned} \alpha _{ \mathbb {C} }\left( d+1\right) -\alpha _{ \mathbb {C} }\left( d\right) =R(d)\text {,} \end{aligned}$$

where *R*(*d*) is a non-trivial combination of integrals of Laguerre functions (see Proposition 1 for the precise expression), reducing the conjecture to showing that R(d)<0. However, the estimation of *R*(*d*), which can be written as the sum of two non-balanced hypergeometric functions, has revealed a highly non-trivial analysis task, and the conjecture remained open for at least 10 years. It is the purpose of this letter to provide the proof of (1.3) in the square case (this can be extended to square Wishart matrices with fixed regularity, see the remarks at the end of the paper and the details in [11]) and to make a brief exposition of the problem, of the real counterpart, and of the origins and some consequences of the conjecture.

The author should be make clear the current limitation of (1.3), which, given the challenges in showing that R(d)<0, is not even close to a proof of the conjecture. Nevertheless, a recent preprint of Ondrej Hutní k [11], personally communicated to the author, presents a delicate analysis argument starting from the (1.3), which seems to have settled the conjecture. This has weighted on the decision to publish this note as a short letter.

The average singular values conjecture can be precisely stated as follows.

#### Conjecture 1

*(Bandeira, Kennedy, Singer, 2016)* Let $\alpha _{ \mathbb {C} }\left( d\right)$ be the average singular value of a complex valued d×d matrix $\frac{1}{\sqrt{d}}X_{d}$, with random i.i.d. $\mathcal {N} \left( 0,1\right)$ entries. Then, for all d>1,

1.4 $$\begin{aligned} \alpha _{ \mathbb {C} }\left( d+1\right) -\alpha _{ \mathbb {C} }\left( d\right) <0\text {.} \end{aligned}$$

Conjecture 1 has immediate applications in the analysis of expected singular values. For instance, *assuming* (1.4), then

$$\begin{aligned} \alpha _{ \mathbb {C} }\left( 1\right) =\sqrt{\frac{\pi }{4}}\text {,} \end{aligned}$$

together with (1.1) provides, for all d≥1,

1.5 $$\begin{aligned} \frac{8}{3\pi }<\alpha _{ \mathbb {C} }\left( d\right) \le \sqrt{\frac{\pi }{4}}\text {.} \end{aligned}$$

This would improve, in particular, the lower bound $\frac{8}{3\pi }-\frac{ 5.05}{d}\le \alpha _{ \mathbb {C} }\left( d\right)$ from [4, Theorem 7].

### The recurrence relation for the average singular value

First define the constants

1.6 $$\begin{aligned} \left\{ \begin{array}{c} \delta _{d}=(d+1)^{-\frac{1}{2}}-d^{-\frac{1}{2}}<0 \\ \widetilde{\delta }_{d}=(d+1)^{-\frac{3}{2}}-d^{-\frac{3}{2}}<0 \end{array} \right. \end{aligned}$$

The precise statement follows.

#### Proposition 1

Let $\alpha _{ \mathbb {C} }\left( d\right)$ be the average singular value of a Gaussian complex valued d×d matrix $\frac{1}{\sqrt{d}}X$, with random i.i.d., $\mathcal {N}\left( 0,1\right)$ entries. Then, for all d≥1,

1.7 $$\begin{aligned} \alpha _{ \mathbb {C} }\left( d+1\right) -\alpha _{ \mathbb {C} }\left( d\right) =R(d)\text {,} \end{aligned}$$

where

1.8 $$\begin{aligned} R(d)=\delta _{d}\int _{0}^{\infty }x^{\frac{1}{2}}e^{-x}L_{d}(x)^{2}dx+ \widetilde{\delta }_{d}\int _{0}^{\infty }x^{\frac{3}{2} }e^{-x}L_{d}^{(1)}(x)L_{d-1}^{(1)}(x)dx\text {.} \end{aligned}$$

The Laguerre polynomials involved in *R*(*d*) are defined, for general parameter $\alpha \ge 0$, by

$$\begin{aligned} L_{d}^{\alpha }(x)=\sum _{k=0}^{d}(-1)^{k}\left( {\begin{array}{c}d+\alpha \\ d-k\end{array}}\right) \frac{x^{k}}{ k!}\text {.} \end{aligned}$$

The recurrence in Proposition 1 reduces the proof of Conjecture 1 to showing that

1.9 $$\begin{aligned} R(d)<0\text {.} \end{aligned}$$

Proving (1.9) is non-trivial and requires a delicate term by term comparison of the expansion of the integrals in (1.8). The involved integrals can be written as hypergeometric $_{3}F_{2}$ functions, but they are not well-poised and do not admit relevant simplification; the author has tried to compare the two expansions in (1.8) term by term, but this approach was not successful in delivering the aimed conclusion. The estimations required are further complicated because the negativity is not dominated by the largest term of the expansion. State of the art symbolic proof packages, as Kauers’ SumCracker, which has provided several symbolic proofs involving sums (for instance, the positivity of the final expression in [2]), were not able to settle (1.9). It should be emphasized that the expression inside the integrals has changes of signs. Thus, the singular values intensity can only be decreasing in average. The approach in [11] starts by expanding the integrands in (1.8) into series of Laguerre polynomials $L_{d}^{\frac{1}{2}}(x)$, in order to take advantage of their orthogonality with respect to the weight $x^{\frac{1 }{2}}e^{-x}$. The expression is then rewritten by observing a telescoping structure in the tail and it is observed that the leading contributions are not enough to provide the required bounds, which must be sharp. A delicate analysis of all the terms is then performed. The analysis, involving elliptic integrals and Wallis’s product estimates, lead to sharp lower bounds that settle the problem for d>7. Then the cases $1\le d\le 7$ are computed exactly. The smallness of these exact computations already hint that the estimates required to settle the problem must be extremely precise.

### The real case

In [4] it is also conjectured that the average singular value $\alpha _{ \mathbb {C} }\left( d\right)$ of a *real* random matrix is monotonic, but that it *increases* with dimension, rather than decreasing, as we have shown to be the case for complex matrices. The problem is likely to be more difficult, because we are dealing with a β-ensemble with β=1 and the factor

$$\begin{aligned} \prod _{j,k}\left| \lambda _{k}-\lambda _{j}\right| ^{\beta } \end{aligned}$$

leads to eigenvalue intensities given by Pfaffians (see, for instance, [10] or [7]), which are far more complicated to deal in comparison with the case β=2, which leads to a determinantal point process structure. Still, Conjecture 1 provides information for the real case. In [4, Lemma 21] it has been proved that

1.10 $$\begin{aligned} \frac{8}{3\pi }-\frac{9.07}{d}\le \alpha _{\mathbb {R}}\left( d\right) \end{aligned}$$

and that

$$\begin{aligned} \alpha _{ \mathbb {C} }\left( d\right) -\alpha _{\mathbb {R}}\left( d\right) \le \frac{4.02}{d} \text {.} \end{aligned}$$

Combining this last inequality with (1.5) leads to the following improvement of (1.10):

$$\begin{aligned} \frac{8}{3\pi }-\frac{4.02}{d}\le \alpha _{\mathbb {R}}\left( d\right) \text { .} \end{aligned}$$

### The little Grothendieck problem over the unitary group

Conjecture 1 has an immediate application to *the little Grothendieck problem *over the unitary group $\mathcal {U}_{d}$ ($U\in \mathcal {U}_{d}$ if and only if $UU^{H}=U^{H}U=I_{d\times d}$), the problem considered in [4] that originally motivated the conjecture: given $C\in \mathbb {C}^{dn\times dn}$ a complex valued semidefinite matrix, find

$$\begin{aligned} \max _{U_{1},...,U_{n}\in \mathcal {U}_{d}}\sum _{i=1}^{n}\sum _{j=1}^{n}tr \left( C_{ij}^{H}U_{i}U_{j}^{H}\right) \text {.} \end{aligned}$$

In [4], an algorithm called *Orthogonal-Cut* for solving the above problem is presented, together with the following bounds.

#### Theorem 1

[4] Let C⪰0 and complex. If $W_{1},...,W_{n}\in \mathcal {U} _{d}$ are the random output of the Unitary version of the Orthogonal-cut algorithm, then

$$\begin{aligned} \mathbb {E}\left[ \sum _{i=1}^{n}\sum _{j=1}^{n}tr\left( C_{ij}^{H}W_{i}W_{j}^{H}\right) \right] \ge \alpha _{\mathbb {C} }(d)^{2}\max _{U_{1},...,U_{n}\in \mathcal {U}_{d}}\sum _{i=1}^{n} \sum _{j=1}^{n}tr\left( C_{ij}^{H}U_{i}U_{j}^{H}\right) \text {.} \end{aligned}$$

#### Remark 1

Combining this with Corollary 1 leads to the *d*-independent inequality

$$\begin{aligned} \mathbb {E}\left[ \sum _{i=1}^{n}\sum _{j=1}^{n}tr\left( C_{ij}^{H}W_{i}W_{j}^{H}\right) \right] \ge \left( \frac{8}{3\pi }\right) ^{2}\max _{U_{1},...,U_{n}\in \mathcal {U}_{d}}\sum _{i=1}^{n}\sum _{j=1}^{n}tr \left( C_{ij}^{H}U_{i}U_{j}^{H}\right) \text {.} \end{aligned}$$

#### Remark 2

Conjecture 1 shows, as suggested in [4], that the approximation ratio in the algorithm gets worse as the dimension increases.

### Turán determinants

Proposition 1 follows from a connection with Turán determinants. The *Turán determinant* of a sequence of orthogonal polynomials $\{P_{n}\}$ is an expression of the form

1.11 $$\begin{aligned} \left| \begin{array}{cc} P_{n}(x) &  P_{n-1}(x) \\ P_{n+1}(x) &  P_{n}(x) \end{array} \right| =P_{n}(x)^{2}-P_{n+1}(x)P_{n-1}(x)\text {.} \end{aligned}$$

The following identity shows that the polynomial part of the singular values intensity of the Laguerre ensemble of order 0 (given in terms of $$ L_{n}(x)=L_{n}^{(0)}(x)$$) can be written as a Turán determinant in terms of the Laguerre polynomials $$L_{d}^{(1)}(x)$$:

$$\begin{aligned} d^{\frac{1}{2}}\Delta _{d}(x)=L_{d-1}^{(1)}(x)^{2}-L_{d-2}^{(1)}(x)L_{d}^{(1)}(x)\text {.} \end{aligned}$$

Turán [14] proved that the expression (1.11) is positive in the case of Legendre polynomials. In [13] Szegö found a manifold of connections with other mathematical areas which lead to several different proofs of (1.11). This is still an active research topic (see, e. g., [5] for hypergeometric functions, where some conjectures are stated). While Turán and Szegö ’s results concern only the positivity of these expressions and are mostly based on analytic methods, our approach is more similar to Szász’s [12], who obtained recurrence relations for Turán expressions.

## Proof of Proposition 1

The average singular value $\alpha _{\mathbb {C} }\left( d\right)$ can be expressed in terms of Laguerre polynomials. The singular values of $\frac{1}{\sqrt{d}}X_{d}$ are the square-roots of the eigenvalues of the Wishart matrix $\frac{1}{d}W_{d}$. The eigenvalue distribution of such eigenvalues in the bulk of the spectrum is (see [16, Theorem 2.17], [7] or [8]),

2.1 $$\begin{aligned} \frac{1}{d}\sum _{n=0}^{d-1}e^{-x}L_{n}(x)^{2}{, \ \ \ }x\ge 0\text {,} \end{aligned}$$

where $L_{n}(x)=L_{n}^{(0)}(x)$ is the Laguerre polynomial, defined, for general parameter $\alpha \ge 0$, as

$$\begin{aligned} L_{n}^{(\alpha )}(x)=\sum _{k=0}^{n}(-1)^{k}\left( {\begin{array}{c}n+\alpha \\ n-k\end{array}}\right) \frac{x^{k} }{k!}\text {.} \end{aligned}$$

By combining the above definitions, one can write $\alpha _{ \mathbb {C} }\left( d\right)$ explicitly as

2.2 $$\begin{aligned} \alpha _{ \mathbb {C} }\left( d\right) =\int _{0}^{\infty }x^{\frac{1}{2}}e^{-x}\Delta _{d}(x)dx \text {,} \end{aligned}$$

where

$$\begin{aligned} \Delta _{d}(x)=\frac{1}{d^{\frac{3}{2}}}\sum _{n=0}^{d-1}L_{n}(x)^{2}\text {.} \end{aligned}$$

Now, the *Christoffel-Darboux formula* tells [3, Theorem 5.2.4s]:

2.3 $$\begin{aligned} \frac{1}{d}\sum _{n=0}^{d-1}L_{n}(x)L_{n}(y)=\frac{ L_{d}(x)L_{d-1}(y)-L_{d-1}(x)L_{d}(y)}{x-y}\text {.} \end{aligned}$$

Setting x=y in (2.3) we obtain

$$\begin{aligned} d^{\frac{1}{2}}\Delta _{d}(x)=\frac{1}{d} \sum _{n=0}^{d-1}L_{n}(x)^{2}=L_{d-1}(x)\frac{d}{dx}L_{d}(x)-L_{d}(x)\frac{d}{ dx}L_{d-1}(x)\text {.} \end{aligned}$$

Using the formulas [3, (6.2.16), (6.2.18)]

2.4 $$\begin{aligned} \left\{ \begin{array}{c} \frac{d}{dx}L_{d}(x)=-L_{d-1}^{(1)}(x) \\ L_{d}(x)=L_{d}^{(1)}(x)-L_{d-1}^{(1)}(x) \end{array} \right. \text {,} \end{aligned}$$

then $d^{\frac{1}{2}}\Delta _{d}(x)$ becomes *a Turán determinant for *$L_{d}^{(1)}(x)$, the Laguerre polynomials of parameter α=1:

2.5 $$\begin{aligned} d^{\frac{1}{2}}\Delta _{d}(x)=L_{d-1}^{(1)}(x)^{2}-L_{d-2}^{(1)}(x)L_{d-2}^{(1)}(x)\text {.} \end{aligned}$$

Now, set

$$\begin{aligned} P=L_{d}^{(1)}(x){, \ \ \ \ \ \ \ \ }Q=L_{d-1}^{(1)}(x) \end{aligned}$$

and use the recurrence formula [3, (6.2.5)]

2.6 $$\begin{aligned} L_{d+1}^{(1)}(x)=\left( 2-\frac{x}{d+1}\right) L_{d}^{(1)}(x)-L_{d-1}^{(1)}(x)\text {,} \end{aligned}$$

to eliminate $L_{d+1}^{(1)}$ from $\Delta _{d+1}(x)$, leading to

2.7 $$\begin{aligned} (d+1)^{\frac{1}{2}}\Delta _{d+1}(x)=P^{2}+Q^{2}-\left( 2-\frac{x}{d+1} \right) PQ\text {.} \end{aligned}$$

Then replace d→d-1 in (2.6) to yield

$$\begin{aligned} L_{d}^{(1)}(x)=\left( 2-\frac{x}{d}\right) L_{d-1}^{(1)}(x)-L_{d}^{(1)}(x) \text {,} \end{aligned}$$

which allows to eliminate $L_{d-2}^{(1)}$ from $\Delta _{d}(x)$:

2.8 $$\begin{aligned} d^{\frac{1}{2}}\Delta _{d}(x)=P^{2}+Q^{2}-\left( 2-\frac{x}{d}\right) PQ \text {.} \end{aligned}$$

Subtracting (2.8) from (2.7) we obtain a recurrence relation for $\Delta _{d}(x)$ which can be considerably simplified as follows

2.9 $$\begin{aligned}  &   \Delta _{d+1}(x)-\Delta _{d}(x) \nonumber \\  &   \quad =\left( (d+1)^{-\frac{1}{2}}-d^{-\frac{1}{2}}\right) \left( P^{2}+Q(x)^{2}\right) \nonumber \\  &   \qquad -\left[ (d+1)^{-\frac{1}{2}}\left( 2-\frac{x}{d+1} \right) -d^{-\frac{1}{2}}\left( 2-\frac{x}{d}\right) \right] PQ \nonumber \\  &   \quad =\left( (d+1)^{-\frac{1}{2}}-d^{-\frac{1}{2}}\right) \left( P^{2}+Q^{2}-2PQ\right) \nonumber \\  &   \qquad -\left( d^{-\frac{3}{2}}-(d+1)^{-\frac{3}{2}}\right) xPQ\text {.} \end{aligned}$$

Recognizing the factor $\left( P-Q\right) ^{2}$ in (2.9) and using the definition (1.6) of the constants $\delta _{d}$ and $\widetilde{\delta }_{d}$, we arrive to

$$\begin{aligned} \Delta _{d+1}(x)-\Delta _{d}(x)=\delta _{d}\left( P-Q\right) ^{2}+\widetilde{ \delta }_{d}xPQ\text {.} \end{aligned}$$

From the second equation in (2.4), $L_{d}(x)=P-Q$ and we conclude that

$$\begin{aligned} \Delta _{d+1}(x)-\Delta _{d}(x)=\delta _{d}L_{d}(x)^{2}+\widetilde{\delta } _{d}xL_{d}^{(1)}(x)L_{d-1}^{(1)}(x)\text {.} \end{aligned}$$

Finally, due to (2.2), integration against $x^{\frac{1}{2}}e^{-x}$ gives the result.

### Remark 3

It is possible to extend Proposition 1 in order to obtain a recurrence relation for $\alpha _{ \mathbb {C} }^{(\alpha )}\left( d\right)$, defined by integrating

2.10 $$\begin{aligned} \Delta _{N}^{(\alpha )}(x)=\frac{1}{N^{\frac{3}{2}}}\sum _{n=0}^{N-1}\frac{ \Gamma (n+1)}{\Gamma (n+\alpha +1)}L_{n}^{(\alpha )}(x)^{2} \end{aligned}$$

against $x^{\alpha +\frac{1}{2}}e^{-x}$. See the details in [11], where, setting α=M the resulting recurrence relation has been used to extend the monotonicity of the average singular value of a complex rectangular *N* ×(N +M) Wishart matrix, when *M* remains fixed and *N* grows..

## Data Availability

No datasets were generated or analysed during the current study.
